# Supplementary material for: Acceptability of the voice your values, an advance care planning intervention in persons living with mild dementia using videoconferencing technology
Source: PLoS One. 2022 Apr 26;17(4):e0266826. doi: 10.1371/journal.pone.0266826 (PMC9041841; doi:10.1371/journal.pone.0266826)
Supplement: S1 Table — (DOCX) [file pone.0266826.s001.docx]

**S1 Table*.* Categories and Subcategories Related to the Acceptability of VYV with Additional Quotes**

| Categories | Subcategories | Description of Subcategories | Additional Illustrative Quotes |
| --- | --- | --- | --- |
| Acceptability:  5 categories were established to describe the acceptability of VYV in persons with mild dementia and their trusted individuals | - Breaking the ice | - VYV as a means to engage in ACP process | OA: "I do have a problem with my values and what I want because when it’s the end, it doesn’t matter what I think, but I realize it will help them make the decision, and so the decision is a little easier. And having it written down on a paper, might even be better, so you can look at it"  OA: “Well at first, I didn’t think it was that important. But, after going through this with SV I realize how important it is, so that if it’s not my wife, like it could be my daughter that has to make the decision, the decisions that would have to be made would be easier if she had a plan in front of her to look at. And that’s basically what I realized is important, which I didn’t before. Yeah before I thought a person could make up their own mind it doesn’t matter, but I think it would help if I had it written down.”  TI : “Never had a discussion about her values and wishes with anyone, as she lives in the present, this is probably the first time we are having this discussion.”  TI: “It allows the person with dementia to open up a bit, when otherwise she probably would not have. It's something that I know she is not comfortable talking about. But having the voice your values session allowed her to open up, and progression can be seen from first time to now. At the beginning she was sort of reluctant and not too happy we were doing it, but in the latter stages she seemed more positive in her answers and seemed to be enjoying herself.” |
|  | - Revealing the values and wishes for future care | - VYV as a medium for PLwD* to express values and wishes and for trusted individuals to learn about them | OA: “I will/may become no longer me. Then I would say shove me in a home, go on with your life. And don’t feel any guilt. That’s the thing, you go on an don’t feel any guilt.”  OA: "There are the basics and there are the heroics, I don’t want heroics, I would rather go than suffer for 6 months and I don’t want my wife to go through this for 6 months"  TI 17: “I think one of the things that came out of this meeting (VYV) was that I had made decisions on my own without really consulting her. Whereas, because of our interaction with SV, I found out what she was thinking."  TI: “I would say that I have always thought about being there and taking care of him in any health level, but I never thought it would get to this level. He is much more dependent on me. I have known him to be a very strong person and father. I always thought that he would take care of himself and he could take care of himself, but to see him in this situation, it kind of rattles me and confuses me. And I kind of also lost hope and was angry, but this study and the idea of advanced care planning, kind of let me know that as bad as the situation is it is not unmanageable. But it helped me get a grip on the reality of the situation. So, it did help during the interview with SV, we were together and he was answering the questions. He was also forced to think about it which is not something he thinks about all the time. And I was able to get some insight on his thinking and opinions, which was great. It helped him express himself. It helped him by at least by telling me, because I had never verbally heard his values and his desires, but in the back of my mind I already had an idea, but it was good to hear.” |
|  | - Getting all the cards on the table | - VYV as an opportunity for dialogue among participants | OA: “I don’t want to die like mother did, oh God. Okay, let’s try … should I get cancer or diagnosed with cancer. If this thing turns out to be throat cancer or stomach cancer I will not want to go through radiation or whatever. Just keep me comfortable, thank you very much. And I already have a disease that will be shear hell to my family and myself. To see my brain, to not be able to converse with my wonderful children, and husband and grandchildren. To not understand them, to be lying there in my own excrements to be wearing my brassiere over top of my clothing, to start ripping up my family’s pictures, to be eating artificial flowers – all of which happened to my mother. I don’t want to put my family through that. So, I would rather them watch me decline with cancer than watch me decline and eventually die with Alzheimer’s. And it's very hard because that would go against everybody I think, because right now I am still me more or less.”  TI: “It (VYV) allowed both of us to state how we felt and to come to the conclusion of what he wanted and my support of it.”  TI: “It is part of the overall coming to grips and generating discussions between the two of us. Which in the near future will generate conversation with our two children, so that we are sort of all on the same page and we understand each other's positions. So there will be a family briefing on what was talked about once we get the hard copy and review it ourselves.” |
|  | - Coming to grips | - VYV as a tool for the trusted individuals to prepare for future decision making | TI: “She (SV) helped me to become more focused and balanced, and to realize what I was going through is normal and don’t be hard on myself. I was grateful for that. And it helped me and will help me to see things that mom will go through and the importance of having things in place in the event she reaches the stage that she cannot speak for herself.”  TI: “It kind of just opened our eyes because we realized we don’t have anybody else. So not thinking of myself and what might happen to me, we have to think about that. It’s very important. We decided it is something we actually have to do is put everybody in their place so we know what’s happening with everybody if one person, like if our pyramid goes down how are we going to fix it. I think it’s helped us realize that we do have to plan for these things and not just hope for tomorrow to be a better day, more or less. It opened our eyes a lot, but it’s almost kind of depressing that I’m at that age where I have to think that way, you know?”  OA: "I dont want to suffer, at the same time I dont want my family to see me suffer, I could say leave it alone, let god do the thing" - Appeared conflicted as she wants god to take her decisions, and be comfortable, but at the same time she does not want her family to live without her and she does not want them to see her suffer too. - "I dont want to see them go down to this kind of environment (talking about hospital or a nursing home), at the same time, I dont want to be in too much pain myself, it is very tough" |
|  | - A third person to bounce it off | - Value in presence of knowledgeable facilitator/clinician | OA: “Specially trained people who zero in on these situations are much more prepared for the answers and the non-answers that are coming from the family members than dealing with a nurse practitioner or doctor that you see probably four times the last 10 years. I think that this is a better way of doing it, so that everybody's on the same page.”  TI: “I think it's very necessary to have somebody who understands the interplay between dementia and the physical issues and some of the potential scenarios. And the willingness to be able to talk about some very tough situations and challenges with folks who may tend to kind of veer off a little bit”  TI: “Well I found it very helpful to really solidify his wishes and we talked about some scenarios that even though we had talked about it before we hadn’t come up with those specific kind of situations. So there was a lot of clarity around some things that we hadn't really been considered so I think it was very positive.” |
| Participants’ experiences of engaging in VYV virtually | - Opinions about videoconferencing |  | OA: “Over the computer is good, because you feel like the person is almost right there with you. Over the phone it can feel like they are a million miles away.”  OA: “I am not really skilled in this…but I am very willing and am able with my husband’s help and yours to make the best of things.”  TI: “If there was no COVID, of course in person would probably have been better. It is always better to see the person.”  TI: “I was actually surprised at how good the online was.”  TI: “Sometimes in person can also be difficult because some of the questions were more difficult to answer and were easier to answer virtually than to answer to somebody in person, at least I found for myself” |
|  | - Challenges with videoconferencing |  | OA: "Covid has made it so much harder, the fact that we have to do it over the computer, instead of sitting across from each other has made it so much harder, though it was completely successful"  OA: “Well, I do have hearing problems and wear two hearing aids so I am always nervous if I am going to hear you. But I do hear you okay. I guess one of the challenges is it is something new. And the other thing is when I sit face to face with someone you read their faces, you read their eyes and everything else when you are talking to them.”  TI: “The other challenge is I had to physically be here with her, I had to help her set it up.”  TI: “I think in person would have some benefits and some challenges, such as somebody travelling and for people who might be in the early stages of dementia to travel, but at the same time it might be a challenge if they don’t have somebody who has access to a computer and the knowledge to connect. The benefits to in person are that it is easier for both parties to observe facial expression and body language and that type of thing.” |

OA Older adult (Person living with dementia)

TI Trusted individual
