# Supplementary material for: Acceptability of the voice your values, an advance care planning intervention in persons living with mild dementia using videoconferencing technology
Source: PLoS One. 2022 Apr 26;17(4):e0266826. doi: 10.1371/journal.pone.0266826 (PMC9041841; doi:10.1371/journal.pone.0266826)
Supplement: S1 File — (DOCX) [file pone.0266826.s002.docx]

# Voice Your Values, A Tailored Advance Care Planning Intervention in Persons Living with Mild Dementia: A Pilot Study

Shirin Vellani,^[[1]](#footnote-1)^ NP, Ph.D., Martine Puts,^2^ Ph.D., Andrea Iaboni,^3,4^ MD, DPhil , Katherine S. McGilton,^2,3^ Ph.D.

Corresponding author: Shirin Vellani

Faculty of Health Sciences, School of Nursing, Health Sciences Centre, McMaster University, 1280 Main Street West, Hamilton, ON, 2J20L8S 4K1, Canada Tel: 1 647 281 6871.

mail: [shirin.vellani@mail.utoronto.ca](mailto:shirin.vellani@mail.utoronto.ca) Twitter Handle: @vellani_shirin

Word Count: Abstract - 250, Text - 3500

Number of Tables: 3; Number of Figures: 1; Number of Supplementary Files: 3; Number of References: 39

## **Abstract**

Persons living with dementia (PLwD) are able to identify their values and wishes with a high degree of accuracy and reliability in the early stage. However, advance care planning (ACP) is not incorporated in routine dementia care. We developed an intervention called Voice Your Values (VYV) that healthcare professionals can implement to identify and document values of PLwD and their trusted individuals. **Purpose:** This single group pre-test and post-test design aimed to determine the feasibility and preliminary efficacy of the VYV intervention. **Methods:** A convenience sample of 21 dyads of PLwD and their trusted individuals was recruited from five geriatric clinics. The VYV intervention was delivered to the dyads over two sessions using videoconferencing technology. Feasibility was determined based on recruitment and retention rates, and intervention fidelity*.* Preliminary efficacy was examined using an ACP engagement survey to assess the PLwD’s engagement in the ACP process; surrogate decision-making confidence scale and dementia knowledge assessment tool in trusted individuals; and Kessler psychological distress scale in all participants. **Results:** The recruitment rate was 52%; the retention rate was 94%. Intervention fidelity was high based on the audit of 20% of the sessions. PLwD demonstrated improvement in ACP engagement (*p=*<.01); trusted individuals showed improvements in decision-making confidence (*p*=.02) and psychological distress (*p*=.02); whereas no change noted in their dementia knowledge (*p=*.47). **Conclusion:** Most of the feasibility parameters were met. A large sample along with a control group, as well as a longitudinal study, are requisite to rigorously evaluate the efficacy of the VYV intervention.

***Key Words:*** Dementia, advance care planning, community-dwelling older adults, pilot study

## **Key Message**

This article describes a pilot study of a tailored advanced care planning intervention involving persons living with dementia (PLwD) and people they trust. PLwD were keen to express their values and wishes for future care. Results showed increased decision-making confidence in trusted individuals and decreased psychological distress in all participants.

## **Introduction**

There is an increasing acceptance of the terminal nature of dementia amongst clinicians. However, integration of a palliative approach in dementia care is not a standard of practice. Older adults living with advanced dementia often experience acute care admissions including to critical care in the last month of their life and receive poor end-of-life care (1, 2). These life-sustaining treatments do not appear to have a positive impact on the person’s quality of life and reversal of mortality. In the advanced stage, people are generally unable to engage in their own care decisions. Their care partners who are expected to make these decisions, express feeling confused and burdened with the task of proxy decision-making (3). The awareness of an individual’s wishes and their prognosis can guide care partner’s decision-making during medical crises and may reduce their psychological distress (4, 5). Advance care planning (ACP) is a process that allows individuals to exercise their autonomy in preparing others to make informed future care decisions for them (6).

In the early stage of dementia, individuals are able to participate meaningfully in identifying their wishes, values, and goals of care (7, 8) with a high degree of accuracy and reliability (9). However, percentage of individuals living with advanced dementia who have engaged in any ACP discussions varies widely, from 1.4% to 39%, depending on the source (4, 10, 11). In our recent scoping review, we found that primary care practitioners believe they should hold ACP discussions with persons living with dementia (PLwD) and their trusted individuals; but many are not sure when and how to carry out these discussions (12). There is a lack of evidence-informed guidelines to implement ACP discussions for PLwD, and particularly for those in the early stages of the disease. To address this gap, we have designed an intervention called Voice Your Values (VYV) that healthcare professionals can implement in their practice to identify and document the values and wishes of individuals living with mild dementia as a means of engaging them and their trusted individuals, such as friends or family, in the ACP process. The purpose of this study is to assess the feasibility, acceptability, and preliminary efficacy of the VYV intervention in community-dwelling older adults living with mild dementia and their trusted individuals. In this paper, we report on the following specific objectives:

1. Examine the feasibility of implementing the VYV intervention in community-dwelling older adults with mild dementia and their trusted individuals as measured by recruitment and retention rates and intervention fidelity.
2. Identify the influence of the VYV intervention on trusted individuals’ level of decision-making confidence, dementia knowledge and psychological distress when compared to their baseline levels.
3. Determine the effect of the VYV on PLwD’s level of engagement in the ACP process; and psychological distress when compared to their baseline levels.

## **Methods**

#### **Design**

This is a single-group pre-test and post-test design (Supplemental file 1. CONSERVE checklist) (13). The pre-pandemic plan was to conduct a randomized feasibility trial. However, refusing an ACP intervention in COVID-19 era was considered unethical given a higher number infection and adverse outcomes were seen in older adults. The study received ethics approval from the University Health Network Research Ethics Board, University of Toronto, as well as the five recruitment sites. The template for intervention description and replication (TIDieR) checklist (14) was used to describe details of the VYV intervention for future replication (Supplemental file 2).

#### **Participants**

Participants were English-speaking community-dwelling older adults 65 years and older who had a diagnosis of mild dementia, as diagnosed by their physician or nurse practitioner. They had access to a device with internet connection for videoconferencing and a trusted individual with at least weekly contact who could enroll in the study. PLwD were not eligible if they had an acute psychotic disorder, and/or clinical depression, due to concerns that these conditions could limit their ability to participate meaningfully in ACP discussions; blindness or deafness; or if they already had a written advance care plan in place. A written advance care plan was any document that identifies a person’s values, wishes and/or future goals of care. Living wills, power of attorney and financial planning documents were not considered ACP documents. English-speaking trusted individuals 18 years and older were included, who did not have dementia, blindness, or deafness and had access to a device for videoconferencing.

#### **Consent**

Participants were recruited from five geriatric clinics situated in geographically diverse locations in Ontario, Canada. Recruitment and data collection lasted between July 2020 and February 2021. This was the most trying times in the COVID-19 pandemic, as vaccines were still not available. Eligible participants were identified by a physician or a nurse practitioner at the clinics. Enhanced consenting techniques were used (15) for obtaining verbal consent and participants’ comprehension was assessed using the teach-back method (17) by the PI (SV). Due to the pandemic related physical distancing measures, all study procedures were completed over the Health Information Protection Act, 2016 (16) compliant, Microsoft (MS) Teams videoconferencing platform.

#### **The Voice Your Values Intervention**

The VYV intervention was developed based on empirical data and the theoretical underpinnings of the Representational Approach to Patient Education (18-20) and the Transtheoretical Model of Stages of Change (21), while the process of delivering it was guided by the recommendations of Piers et al. (22). The Representational Approach asserts that effective education can only happen if patients are given the opportunity to share their knowledge and beliefs about the matter on hand, i.e., dementia and ACP, before they are provided with new information. The Transtheoretical Model suggests that people move through six stages of change to acquire a new behavior (21). ACP is considered to be a complex process and change in behaviors is dependent on several processes of change (21, 23). These processes can be positively influenced by the presence of an interventionist (PI) with knowledge and experience in the care of older adults in turn help individuals to move forward on the ACP trajectory. In terms of carrying out the ACP discussion (domain iii), which is the core of the VYV intervention, its essential elements included: 1) education to increase dementia knowledge, 2) establish the need for ACP, and 3) personalized coaching to identify future care values and wishes of PLwD. The VYV intervention was a tailored ACP intervention delivered over two sessions and involved one-on-one interactions between the dyads and the interventionist (PI). Though the initial plan involved an in-person delivery, due the ongoing pandemic it was modified to be delivered via videoconferencing (See Table 1 for the

#### **Measures**

Sociodemographic data were acquired from all participants. The Charlson Comorbidity Index (25) was based on past medical history obtained from the referring clinics, that also provided the most recent cognitive test scores. In order to confirm the dementia stage, the Quick Dementia Rating System (QDRS) score was acquired from the trusted individuals (26), to prevent distress that may be caused by cognitive testing in PLwD.

***Assessment of Feasibility***

Feasibility was determined based on the 1) recruitment rate, percentage of enrolled dyads out of the total number of eligible participants invited; 2) retention rate, percentage of consenting dyads who completed all VYV study procedures; and 3) intervention fidelity based on the intervention fidelity checklist completed by the interventionist after each session. Another research staff audited 20% of both the sessions by crosschecking the session video recordings against the fidelity checklist.

***Preliminary Efficacy***

Five outcomes were measured to determine the preliminary efficacy of the VYV intervention in PLwD and their trusted individuals at baseline (T1) and 7-14 days post intervention (T2). Based on previous studies, this timeframe was chosen to minimize the risk of bias that may occur due to any acute changes in condition of the PLwD that may hamper their ability to participate in post-intervention data collection (27). The measures included a 15-item ACP engagement survey that had two sub-scales of the ACP processes including self-efficacy and readiness in older adults (27). The Cronbach alpha was 0.76 indicating acceptable internal consistency of this measure. The responses on the readiness items were used to categorize participants in various stages of behavior change (27). Other outcomes included 5-item Decision-Making Confidence (DMC) scale (28), that demonstrated excellent internal consistency in the current study (Cronbach alpha 0.88), and a 21-item Dementia Knowledge Assessment Tool (29), demonstrating high internal consistency with a Cronbach alpha coefficient of 0.979, in trusted individuals. Psychological distress in all participants was measured using the 10-item Kessler Psychological Distress Scale (K-10) (30, 31). K-10 also showed a high internal consistency in this study with a Cronbach alpha of 0.88 for PLwD and 0.86 for trusted individuals.

#### **Statistical Analysis and Sample Size Estimation**

Statistical analyses were conducted using the SPSS IBM Statistical Software version 27.0 with an α = <0.05. Results are expressed as percentages, mean and standard deviation (SD). A paired-sample *t* test was conducted to compare the mean score on all measures before and after the VYV intervention and change in outcome measures (difference, 95% confidence interval). Preliminary effect sizes of changes were calculated. The McNemar test was used to identify the percentage of participants in the pre-contemplative phase of behavior change at baseline and those who moved forward in the behavior change using the readiness subscale of the ACP Engagement Survey. As this was a feasibility study, no sample size was statistically calculated. However, a 60% recruitment rate was targeted given the virtual mode of delivery requiring no travel for the participants. Due to the COVID-19 pandemic, clinics were functioning at a reduced capacity due to leadership changes, staff redeployment to other areas and additional mandatory measures to prevent the spread of the coronavirus, resulting in lower-than-expected referral rates.

## **Results**

#### **Characteristics of Participants**

Sociodemographic and clinical characteristics of the participants are summarized in Table 2. There were an equal number of male and female PLwD, who were mostly white (n = 11, 55%) followed by South Asians (n = 5, 25%). Mean age of the PLwD was 80 ± 6.6 years. On average, they were diagnosed with dementia 1.8 ± 1.2 years before enrollment. In terms of trusted individuals, they were mostly females (n = 13, 65%) and children of the PLwD (n = 11, 55%), and the majority had contact with the PLwD at least once daily (n= 14, 70%).

#### **Feasibility**

***Recruitment and Retention Rates***

Altogether, the five recruitment sites referred 47 potential participants with a recruitment rate of 52%. Seven individuals were ineligible, and twenty one dyads were recruited over an 8-month period. (See Figure 1 for the CONSORT flow diagram listing the reasons for refusal). Retention rate was high at 94%, with 18 dyads completing all study procedures, and 100% (n= 20) attending both the intervention sessions. Twenty trusted individuals completed all study procedures. One PLwD declined to participate on the day of the baseline data collection. One PLwD missed post-intervention data collection appointment, and another declined to complete one of the measures at post-test due to feeling overwhelmed.

***Intervention Fidelity***

The comparison of the intervention fidelity checklist scores showed full agreement between the PI and the other research staff. On average, each VYV session one ranged between 33-63 minutes, rather than the planned 45-60 minutes. Two dyads took two sittings to complete this session due to the PLwD feeling fatigued. The VYV session two ranged between 47-100 minutes rather than the planned 60-90 minutes. For three dyads, the second session was held over two appointments due to the feeling of tiredness in PLwD. Duration of the sessions did not differ based on the last cognitive test scores or the time since dementia was diagnosed.

#### **Preliminary Efficacy Outcomes**

The VYV intervention demonstrated a significant improvement in the decision-making confidence scores from baseline to post-intervention in trusted individual. There was a significant difference in the mean scores of PLwD on the ACP engagement survey. 55% (n=11) of the PLwD were in the pre-contemplative stage of behavior change at T1, which decreased to 40% (n=8) post intervention, showing upward movement in the behavior change stage, though it was not statistically significant (*p* = .32). In terms of the impact on psychological distress, PLwD had a non-statistically significant decrease in the mean scores of K-10 from baseline whereas the trusted individuals had a significant improvement in their scores. The scores related to dementia knowledge in trusted individuals stayed about the same post-intervention. Six of the 20 PLwD did not remember the partial or full content of the VYV sessions at the time of the outcome data collection. (See Table 3 for Change in Outcomes Post VYV).

## **Discussion**

The VYV tailored ACP intervention demonstrated evidence of feasibility in community-dwelling older adults living with mild dementia and their trusted individuals as evidenced by a high retention rate and a high degree of concurrence between the interventionist and the RA on the intervention fidelity audit. However, some of the intervention sessions took longer than expected to deliver and the recruitment rate was 8% less than the expected. In terms of the preliminary efficacy, our findings demonstrated that the VYV intervention led to significant improvement in the decision-making confidence and psychological distress in trusted individuals. No improvement was seen in their dementia knowledge which is likely due to tailored education rather than following a specific curriculum tested on the selected tool.

One of the indicators of feasibility of the VYV intervention was intervention fidelity that involved conforming to the elements of the VYV sessions laid out in the intervention manual. The interventionist was able to follow the intervention components as planned, and this was confirmed through the audit of 20% of the sessions. However, the time taken to complete the sessions was wide-ranging, with some sessions taking longer than expected. Also, three dyads completed the intervention in three to four sittings rather than the planned two. Longer sessions may raise concerns about the feasibility of the VYV intervention in clinical practice and/or future trials. However, similar observations have been made in another ACP intervention study where it took an average of 100 minutes for a single-session intervention delivered to PLwD instead of 82 minutes when delivered to other older individuals (28). The authors suspected that the lengthier sessions were due to PLwD taking longer to understand questions and retrieve information from their memory to respond. The authors did not recommend breaking the sessions into more than one due to limited memory in PLwD (28). Given that there are no standard guidelines on how best to engage PLwD and their trusted individuals in ACP, there are opportunities to test various options related to the number of sessions and the time required for their delivery. It is important to bear in mind that ACP is a process that cannot be completed in one session regardless of the diagnosis of the person (32). Furthermore, ACP conversations can be emotionally charged for many, requiring sufficient time to prepare for the interventionists and the participants (33).

What appears critical is taking a person-centred approach to ACP given the unique characteristics and circumstances of PLwD. Cognitive and behavioral strategies that help reduce cognitive burden in PLwD are promising (34). Previous studies have also shown enhanced communication strategies such as candid discussion, shared decision-making, and assessing readiness for change and empathy, to be critical to the higher uptake of ACP in older adults (33). The feasibility of conducting ACP sessions in busy clinicians’ offices or virtually requires more research going forward. Teaching interdisciplinary team members to build their knowledge, skills, and competence in the care of older adults and engaging PLwD in the ACP process, is also important going forward.

Our findings are in line with previous ACP studies involving people with mild dementia, that also showed a high retention rate where PLwD meaningfully participated in ACP discussions (28, 35). However, what was unique in the current study was a wide variation seen in the time since participants were diagnosed with dementia, making it difficult to suggest how soon after the diagnosis ACP discussions should be initiated. Nonetheless, each participant had mild dementia and no differences were observed in their level of engagement. Several PLwD displayed some difficulty recalling the full content of the previous sessions. However, each one of them appreciated a summary of the previous session and all were able to articulate their values and wishes for future care with a high degree of clarity. Therefore, it is important not to construe a person’s inability to recall factual information as failure of the ACP intervention. Further research is required to determine if there is an appropriate time for initiating ACP discussions post diagnosis.

In terms of feasibility of recruitment for the VYV intervention, 48% of all referred declined to participate. The most common reason for refusal was related to trusted individuals being unavailable for various reasons. Other reasons expressed by the trusted individuals included fear of being alienated by their PLwD or causing them psychological distress due to the topic of the study, and perception that the PLwD could not engage in ACP discussion due to their diagnosis. Although trusted individuals may be trying to be protective of their PLwD to prevent distress by refusing to participate in the VYV study, these attitudes raise concerns about unknowingly engaging in benevolent ageism (36) while depriving their PLwD of the opportunity to exercise their autonomy. The findings of this study did not demonstrate any increase in PLwD’s level of psychological distress post VYV intervention, so in many cases, this is likely to be an unfounded concern. PLwD were keen to talk about their values and wishes related to care during the terminal and vegetative states. Trusted individuals generally report lack of confidence in decision-making for future care, and lack of knowledge of the PLwD’s wishes can aggravate caregiving burden, as well as lessen the prospect of true person-centred care in the future (37). One of our critical findings was a significant improvement in decision-making confidence in trusted individuals. As such, future studies may also focus on strategies to educate trusted individuals on the importance of participating in ACP.

Compared to other studies, the VYV intervention had some similar features such as structured discussion and strong theoretical underpinnings (28, 35). The VYV study also had several unique features. Firstly, it was conducted by a knowledgeable interventionist who not only prepared tailored education based on each dyad’s needs and gaps in knowledge but was also able to address questions that arose in the moment given her expertise in the care of older adults. To maintain the PLwD’s interest during the intervention, ad hoc activities were incorporated such as singing, stretching, and encouraging them to share interesting anecdotes. These appeared to have a positive impact on rapport building and overall engagement of the PLwD in the ACP process.

In terms of efficacy of ACP, there is no consensus on what the outcomes for ACP studies in PLwD should be (38). While the outcomes were selected for this study based on other ACP trials in older adults, future longitudinal studies are necessary to examine additional meaningful outcomes for individuals with mild dementia. There is need to identify outcomes that do not solely rely on PLwD’s memory. There is also a lack of evidence in the literature on the impact of ACP when conducted in the mild stage on receiving wish concordant care or the receipt of burdensome interventions at the end-of-life (39) such as comfort oral feeding versus enteral nutrition. It is also critical to examine the impact on health system related to early engagement in the ACP such as rate of hospitalizations; admission to intensive care, nursing home and hospice care in the advanced stage; and preferred place of death. There is an important role for clinicians to make concerted efforts to engage PLwD and their trusted individuals in ACP conversations to help normalize these discussions as a necessary part of the care plan. Measures should be taken to increase the capacity of primary care clinicians through education, resource allocation and monitoring indicators of ACP.

## **Limitations**

Though the VYV study provides great insights for future ACP research and clinical initiatives, there are limitations to be considered. There was a small sample size and an absence of a control group. However, it was our intention to focus on a feasibility study and to make considerations for outcome measures to help researchers plan for a larger study to test and refine the elements of ACP in persons living with mild dementia. Given the pre-test post-test design, improvement in the outcomes may be related to repeated testing rather than the effect of the VYV intervention. A larger sample size along with a control group, as well as a longitudinal study, are requisite to rigorously evaluate the effectiveness of the VYV intervention and the outcomes highlighted above.

## **Conclusion**

The current pilot study demonstrated favorable results. While recruitment was challenging, we achieved an acceptable sample size, along with a high retention rate and the intervention fidelity scores. There is promising evidence that people living with mild dementia can effectively participate in ACP to identify their values and wishes for future care.

Disclosures

The authors have no competing interests and conflicts to declare.

Acknowledgements

We would like to thank all the individuals with dementia and their trusted individuals who gracious with their time and efforts to participate in this important project. This research was supported by Maria and Walter Schroeder Institute for Brain Innovation and Recovery.

References

1. Martinsson L, Lundstrom S, Sundelof J. Better quality of end-of-life care for persons with advanced dementia in nursing homes compared to hospitals: a Swedish national register study. BMC Palliat Care. 2020;19(1):135.

2. Houttekier D, Vandervoort A, Van den Block L, van der Steen JT, Vander Stichele R, Deliens L. Hospitalizations of nursing home residents with dementia in the last month of life: results from a nationwide survey. J Palliat Med. 2014;28(9):1110-7.

3. Samsi K, Manthorpe J. Everyday decision-making in dementia: findings from a longitudinal interview study of people with dementia and family carers. Int Psychogeriatr. 2013;25(6):949-61.

4. Vandervoort A, van den Block L, van der Steen JT, Vander Stichele R, Bilsen J, Deliens L, et al. Advance directives and physicians' orders in nursing home residents with dementia in Flanders, Belgium: prevalence and associated outcomes. Int Psychogeriatr. 2012;24(7):1133-43.

5. Hall SPH, Tsouros AD, Costantini M, Higginson IJ. Palliative care for older people: Better practices. Copenhagen: World Health Organization; Regional Office for Europe; 2011. Available from: <https://www.euro.who.int/__data/assets/pdf_file/0017/143153/e95052.pdf>. Accessed on April, 2020.

6. Committee on Approaching Death: Addressing Key End of Life Issues; Institute of Medicine. Dying in America: Improving Quality and Honoring Individual Preferences Near the End of Life. Washington (DC): National Academies Press (US); 2015 Mar 19. PMID: 25927121.

7. Dening KH, Jones L, Sampson EL. Advance care planning for people with dementia: a review. Int Psychogeriatr. 2011;23(10):1535-51.

8. Gregory R, Roked F, Jones L, Patel A. Is the degree of cognitive impairment in patients with Alzheimer's disease related to their capacity to appoint an enduring power of attorney? Age Ageing. 2007;36(5):527-31.

9. Feinberg LF, Whitlatch CJ. Are Persons With Cognitive Impairment Able to State Consistent Choices? Gerontologist. 2001;41(3):374-82.

10. Mitchell SL, Kiely DK, Hamel MB. Dying with advanced dementia in the nursing home. Arch Intern Med. 2004;164(3):321-6.

11. Garand L, Dew MA, Lingler JH, DeKosky ST. Incidence and predictors of advance care planning among persons with cognitive impairment. Am J Geriatr Psychiatry. 2011;19(8):712-20.

12. Vellani S, Puts M, Iaboni A, Degan C, McGilton KS. Integration of a Palliative Approach in the Care of Older Adults with Dementia in Primary Care Settings: A Scoping Review. Can J Aging. 2021 Nov 8:1-17. https://doi.org/10.1017/S0714980821000349. Epub ahead of print. PMID: 34743774.

13. Orkin AM, Gill PJ, Ghersi D, et al. Guidelines for Reporting Trial Protocols and Completed Trials Modified Due to the COVID-19 Pandemic and Other Extenuating Circumstances: The CONSERVE 2021 Statement. JAMA. 2021;326(3):257–265. https://doi.org/10.1001/jama.2021.9941

14. Hoffmann TC, Glasziou PP, Boutron I, et al. Better reporting of interventions: template for intervention description and replication (TIDieR) checklist and guide. BMJ. 2014;348:g1687.

15. Mittal D, Palmer BW, Dunn LB, et al. Comparison of two enhanced consent procedures for patients with mild Alzheimer disease or mild cognitive impairment. Am J Geriatr Psychiatry. 2007;15(2):163-7.

16. Government of Ontario. Health Information Protection Act. 2016. Available from: <https://www.ontario.ca/laws/statute/s16006>. Accessed April, 2020.

17. Cornett S. Assessing and addressing health literacy. OJIN. 2009;14(3):1-.

18. Leventhal H, Nerenz D, Steele, DS. (1984). Illness representations and coping with health threats. In: Baum A, Singer JE, eds. Handbook of psychology and health. New York: Exrlbaum, 1984: 221-52.

19. Leventhal H, Diefenback M. The active side of illness cognition. In: Croyle JASRT, editor. Mental representation in health and illness. New York: Springer-Verlag, 1991: 245-71.

20. Donovan HS, Ward SE, Song MK, et al. An update on the representational approach to patient education. J Nurs Scholarsh. 2007;39(3):259-65.

21. Prochaska JO, Velicer WF. The Transtheoretical Model of Health Behavior Change. Am J Health Promot. 1997;12(1):38-48. https://doi.org/10.4278/0890-1171-12.1.38

22. Piers R, Albers G, Gilissen J, et al. Advance care planning in dementia: recommendations for healthcare professionals. BMC Palliat Care. 2018;17(1):88.

23. Fried TR, Cohen AB, Harris JE, Moreines L. Cognitively Impaired Older Persons' and Caregivers' Perspectives on Dementia-Specific Advance Care Planning. J Am Geriatr Soc. 2021 Apr;69(4):932-937. https://doi.org/10.1111/jgs.16953. Epub 2020 Nov 20. PMID: 33216955; PMCID: PMC8300881.

24. Back AL, Arnold RM, Baile WF, et al. Efficacy of Communication Skills Training for Giving Bad News and Discussing Transitions to Palliative Care. JAMA Intern Med. 2007;167(5):453-60.

25. Charlson ME, Pompei P, Ales KL, MacKenzie CR. A new method of classifying prognostic comorbidity in longitudinal studies: development and validation. J Chronic Dis. 1987;40(5):373-83.

26. Galvin JE. The Quick Dementia Rating System (QDRS): A Rapid Dementia Staging Tool. Alzheimers Dement (Amst). 2015;1(2):249-59.

27. Sudore RL, Stewart AL, Knight SJ, et al. Development and validation of a questionnaire to detect behavior change in multiple advance care planning behaviors. PLoS One. 2013;8(9):e72465.

28. Song M-K, Ward SE, Hepburn K, et al. Can Persons with Dementia Meaningfully Participate in Advance Care Planning Discussions? A Mixed-Methods Study of SPIRIT. J Palliat Med. 2019;22(11):1410-6.

29. Toye C, Lester L, Popescu A, McInerney F, Andrews S, Robinson AL. Dementia Knowledge Assessment Tool Version Two: development of a tool to inform preparation for care planning and delivery in families and care staff. Dementia (London). 2014;13(2):248-56.

30. Bougie E, Arim RG, Kohen DE, Findlay LC. Validation of the 10-item Kessler Psychological Distress Scale (K10) in the 2012 Aboriginal Peoples Survey. Health Rep. 2016 Jan 20;27(1):3-10. PMID: 26788720.

31. Brooks RT, Beard J, Steel Z. Factor structure and interpretation of the K10. Psychol Assess. 2006;18(1):62-70.

32. Levoy K, Salani DA, Buck H. A Systematic Review and Gap Analysis of Advance Care Planning Intervention Components and Outcomes Among Cancer Patients Using the Transtheoretical Model of Health Behavior Change. J Pain Symptom Manage. 2019;57(1):118-39 e6.

33. Frechman E, Dietrich MS, Walden RL, Maxwell CA. Exploring the Uptake of Advance Care Planning in Older Adults: An Integrative Review. J Pain Symptom Manage. 2020 Dec;60(6):1208-1222.e59. https://doi.org/10.1016/j.jpainsymman.2020.06.043. Epub 2020 Jul 6. PMID: 32645455; PMCID: PMC7342022.

34. Kasl-Godley J, Gatz M. Psychosocial interventions for individuals with dementia. Clinical psychology review. 2000;20(6):755-82.

35. Hilgeman MM, Allen RS, Snow AL, Durkin DW, DeCoster J, Burgio LD. Preserving Identity and Planning for Advance Care (PIPAC): preliminary outcomes from a patient-centered intervention for individuals with mild dementia. Aging Ment Health. 2014;18(4):411-24.

36. Vale MT, Bisconti TL, Sublett JF. Benevolent ageism: Attitudes of overaccommodative behavior toward older women. J Social Psych. 2020;160(5):548-58.

37. Maslow K. Person Centered Care for People with Dementia: Opportunities and Challenges. Generations. 2013;37(3):8-15.

38. Wendrich-van Dael A, Bunn F, Lynch J, Pivodic L, Van den Block L, Goodman C. Advance care planning for people living with dementia: An umbrella review of effectiveness and experiences. Int J Nurs Stud. 2020;107:103576.

39. Kelly AJ, Luckett T, Clayton JM, Gabb L, Kochovska S, Agar M. Advance care planning in different settings for people with dementia: A systematic review and narrative synthesis. Palliat Support Care. 2019;17(6):707-19.

1. ^1.^Faculty of Health Sciences, School of Nursing, McMaster University, Hamilton, Ontario, Canada

   ^2.^ Lawrence S. Bloomberg Faculty of Nursing, University of Toronto, Toronto, Ontario, Canada

   ^3.^Toronto Rehabilitation Institute, University Health Network, Toronto, Ontario, Canada

   ^4.^Department of Psychiatry, University of Toronto, Toronto, Ontario, Canada [↑](#footnote-ref-1)
