# Supplementary material for: Acceptability of the voice your values, an advance care planning intervention in persons living with mild dementia using videoconferencing technology
Source: PLoS One. 2022 Apr 26;17(4):e0266826. doi: 10.1371/journal.pone.0266826 (PMC9041841; doi:10.1371/journal.pone.0266826)
Supplement: Attachment — Submitted filename: Dec 20_Response to Reviewer km[14].docx [file pone.0266826.s004.docx]

# Response to Reviewer

# Acceptability of the Voice Your Values, a tailored advance care planning intervention in persons living with mild dementia using videoconferencing technology

Dear Reviewer/s:

Thank you for your thoughtful comments and feedback. We have tried our best to address each of the comment and provide details where requested. Please see the table below for our response in terms of the changes made and corresponding page numbers.

| S. No. | Reviewer’s Comments | Response | Page & Line Numbers |
| --- | --- | --- | --- |
| 1.0 | Abstract  At one place in the abstract, line 18, Voice Your Values has been given VVY as abbreviation. | This typo is now corrected, thank you! |  |
| 2.0 | Method  Important details of the method is missing, see comments below, and for that cause the study is not possible to replicate.  Design  In the design section you state that the feasibility of the intervention is reported on elsewhere, and give the reference for that study. That is good, **but could you please give one short concluding statement about the feasibility in this paper**. Not all readers will bother to search for the referenced article. I presume that the VYV was found feasible otherwise I guess you would not have proceeded with present article. | We have now added the following statement about the feasibility of the study:  ”The findings of the feasibility of the VYV intervention are promising, and the outcomes of preliminary efficacy demonstrated improvement older adults as well as trusted individuals.” | Page 4, line 74 |
| 3.0 | Participants  I wonder if not the interventionist also should be mentioned here? This person is also a kind of participant. I am aware that you write about the interventionist at line 53 but I see that this information rather belongs here since the interventionist participates in the intervention and also evaluates the intervention. | The following statement is added to clarify this:  “The VYV intervention was delivered to participants by an interventionist (PI) who is a trained nurse practitioner with extensive experience working with older adults in a variety of specialized geriatric settings. ” | Page 5, line 92 |
| 4.0  4.1 | Voice Your Values intervention   - In line 104 a researcher named Piers is referenced to for recommendations about how to deliver the intervention but no reference is given for this. I also find no Piers at all in the rest of the manuscript.   Were the interventionist/s working at any of the five participating geriatric clinics? Did the interventionist have any prior relationship to any of the participants through her work? | Thank you for pointing this out, Piers reference is now added in the text and the reference list.  The following statement is now added to the manuscript:  “ The NP interventionist did not work in any of the five participating geriatric clinics .” | Page 6, line 115 |
| 5.0 | Measures  It is not fully clearly stated how the research assistant collected the data. Where this done in a virtual meeting? How was data captured, written down or by audio-recordings? | We have added details on how the measures were completed by the research assistant in the Measures section as follows, displayed in blue:  “Data was collected virtually over MS Teams platform, from all participants 1-2 weeks post intervention session two by a Research Assistant (RA), a registered nurse hired for the study and trained on the measures and methods of virtual data collection. The RA had no prior relationship with any of the participants. During the data collection, the RA displayed the responses for each item/question of the measures using the screen sharing option for the ease of selection. While collecting data, the RA spoke slowly and repeated questions as necessary, making sure questions were adequately comprehended. Data was acquired from each participant of the dyad separately to prevent the influence of the other’s presence on responses. These sessions were video recorded so the RA could provide undivided attention to the participants during the data collection and observe how the respondents were doing in terms of fatigue and distractions. Immediately after the session, RA inputted all the data electronically directly on the UHN drive by viewing the recordings.. .” | Page 6, lines 126-135 |
| 6.0 | Data analysis  In this section the interventionist’s diary log is stated to be used for analysis. Besides from the abstract and at one place in the discussion, is this the only place where the diary log is mentioned. Please add information about the diary log, what was written in it and when under the appropriate heading above in the manuscript (in the methods section). | The following section is added in the measures section:  ”In addition, PI completed a diary after each session that recorded time to complete each session, specific activities carried out, dynamics between the participants, barriers and facilitators; participants’ opinions and perspectives towards VYV, topics of education and any other observations that could help refine the intervention in future trials. This data was then used for triangulation with participants’ qualitative data and helped with intervention fidelity described elsewhere.” | Page 8, lines 169-173 |
| 7.0 | Ethical considerations  It is not clearly stated which **relation the interventionist had** to the participating persons living with dementia. It is neither stated **if the intervention was given as part of a new way of working within the five study sites or if the intervention was only performed as part of the research projec**t. Information about the **research assistant’s relations** to the participating dyads is neither stated. Did this person have a caring relation to the participating person living with dementia or was the research assistant only part of the research group? These issues are important to make clear to understand if there were any power relations to be aware of between the researchers (the interventionist and the research assistant?) and the participants. | The concerns about interventionist’s and research assistant’s relationship are addressed above in points 4.1 and 5.0.  The intervention was performed as part of the research project and not introduced as a part of the new way of working within the five sites. This is now stated in the manuscript as follows in blue:  “The VYV study was completed as a research project where participants were recruited from five geriatric clinics located in geographically diverse regions of Ontario, Canada.” | Page 5, line 96 |
| 7.0  7.1  7.2  7.3  7.4 | Results  The study states to have three research questions:   1. Acceptability of the intervention 2. Factors influencing the acceptability 3. Experiences with video conferencing   As I understand, the quantitative analysis answers the first and the second question but the manuscript states that acceptability is answered through the qualitative analysis. However, I am not convinced about that. TEI is stated to measure acceptability and TEI scores are used for the quantitative analysis. I understand that you had added six open ended questions to the TEI, and as far as I understand the answers to these questions are used in the qualitative analysis. So one part of the acceptability might be answered by your qualitative analysis but one part is answered by the quantitative analysis. **Please adjust the text about which of the research question the different parts of the results are answering.**  The quantitative findings are pretty straightforward reported. However **I struggle with the qualitative findings.** Firstly one question about terminology. In table 4 you state that “Breaking the ice” and all the other “categories” are subcategories but in the text below the table you write about them as categories. At one place you also use the word theme for the results of the qualitative analysis. Please revise for clarity and consistency.  I am also not convinced that the five categories answer any of the questions of the study. I have problems to interpret the five categories as describing “acceptability of the intervention”. Some of them might be possible to understand as a description of acceptability, but “I want to die on my terms” is very hard to interpret as if it deals with acceptability of VYV.  The second category is also formulated so that only the participating persons living with dementias statements can be included. **I would suggest to change the category name in a way that the trusted individual’s views can be included too.**  The qualitative analysis needs to be remade with the research question about “acceptability of the intervention” kept in mind. One other way to sort this out might be to change the research question to fit the analysis that has been made. Maybe the last of the research questions could be rephrased as two? One about experiences of videoconferencing for VYV (which is already answered) and one about what help VYV provides in talking about how one wants to be cared for the last time in life and how one wants to die, or something similar?  For the last part of the results, the part reporting on participants experiences of engaging in VYV virtually is in table 4 stated to be constituted of two sub categories. The categories can be found in the text but in line with how the other equal parts of the results are presented I suggest that the sub categories names are used as headings. | We have now clarified which type of data was collected to address each research question.  Only question number 2 is exclusively answered using the quantitative analysis. Whereas, questions 1 and 3 are explored both quantitatively and qualitatively. This is now clarified by editing the text in the results section in two places, i.e., the beginning of the qualitative findings and the quantitative findings, as follows in blue:  **Quantitative findings**  This section reports on the quantitative data related to the acceptability of the VYV intervention (research question 1), factors influencing the acceptability (research question 2) and the virtual experiences of participants and researcher using the TEI, VEQ-P and VEQ-R questionnaires (research question 3). Of the twenty one PLwD, 18 completed all study procedures…  **Qualitative findings**  In addition to the above quantitative analysis, the qualitative data from the TEI acceptability, VEQ-P, VEQ-R questionnaires and the interventionist diary log provided further insight into the acceptability of the VYV intervention (research questions 1 and 3). Qualitative findings were organized into two broad categories: 1) acceptability of the VYV intervention; and 2) researcher and participants’ experiences with videoconferencing. See table 4 for these categories, respective subcategories, and their description. Table S1 presents additional illustrative quotes for each subcategory.  The text is now edited for clarity and consistency. The first level interpretations are now named categories and the second level, subcategories.  The five subcategories were conceived as describing the acceptability of the VYV intervention. We had identified in the manuscript in lines 533-535 “To ensure acceptability of the VYV intervention, data from open-ended questions and the interventionist diary log were used to better interpret the quantitative data to better understand participants’ views. ” We have now made further clarification by adding the following in blue below.   1) **Acceptability of the VYV Intervention**   While there were some differences between PLwD and trusted individuals in terms of the acceptability of the VYV intervention regarding its content and activities, similar themes were identified between them and thus a combined data analysis was conducted. Five interrelated categories were established that describe the acceptability of the virtually delivered VYV intervention. These include: a) breaking the ice, b) revealing the values and wishes for future care, c) getting all the cards on the table, d) coming to grips, and e) a third person to bounce it off. Details on the categories are presented below. The above categories were conceived as describing the acceptability of the intervention as they demystify some of the myths deterring clinicians to initiate ACP conversations in PLwD. Details on each subcategory are presented below.    Thank you for this suggestion. The name of the second subcategory is now changed to “revealing the values and wishes for future care” to incorporate the views of the trusted individual” and the following statement and quote are now added to the corresponding section:  Inevitably, the VYV intervention provided PLwD an opportunity to think about their wishes and share them, in turn increasing awareness of their trusted individuals about the type of care they desire. This trusted individual expressed:  *It (referring to VYV discussions) just changes a lot of things in terms of the relationship and the future. I think it pinpoints some areas I need to take better care. I think one thing that really stands out is the question about if she was put on life support. It is something that we never discussed, so by her response I learned a lot about what she wanted. I think that particular question was the stand out for me on this.* *(TI 5)*  The subcategories’ names are now identified in the text as headings. | Page 10, lines 212-217  Page 12, lines 237-242  Page 13, lines 252-254  Page 14  Page 15, lines 325-332  Page 18 |
| 8.0  8.1 | Discussion  In the discussion reasons for why VYV was considered acceptable comes forth (lines 389-392). This is the kind of result that I would have expected if acceptability was in focus but the result is not reported in this way.  The discussion also brings forth findings from present study that I do not recognise from the results such as barriers to ACP (lines 425-429). If barriers found through the analysis are going to be discussed they first need to be reported on in the results section. | We have now edited the results section as described previously to bring more clarity and consistency between results and the discussion.  Some of the barriers to the ACP conversations were identified in the qualitative data, this was highlighted in the subcategory, “breaking the ice”. It is further elaborated by adding the following data statement in the same section and thus the results now link with the discussion:  Many believed that their trusted individuals are already aware of their values and wishes while trusted individuals denied having clarity on this and some even shook their heads in disagreement which was captured during the videocall. | Page 13, lines 261-263 |
|  | Strength and limitations  Some limitations of the study are discussed but not the fact that the intervention was performed by the PI of the study. In a future and if the intervention is going to be used as part of daily clinical routines for persons living with dementia the intervention will need to be performed by the regular health care professionals at the geriatric clinics. This fact needs to be discussed not least since the PIs good skills were lifted as a key factor for the acceptability of the intervention. What are your thoughts of the possibility of scaling up the intervention? What potential barriers and possibilities do you see? Do you think that an education will be needed for health professionals to be able to be in charge of the VYV intervention? | Following section in blue is added to provide more insight into potential for incorporating VYV/ACP discussions in routing dementia care:  The ultimate hope is that ACP is embedded in the routine dementia care, so PLwD and their trusted individuals are less apprehensive than if discussions linked to poor prognosis or lack of treatment options. The findings of this study are promising to support the appropriateness of ACP in early dementia. The VYV intervention can be scaled up through measures to upskill the health care professionals including those in the primary care practices through education and resource allocation. One of the components of the VYV intervention is education to the participants and the ability to respond to questions that arise in the moment. Therefore, any clinician who has training in the care of older adults and how to broach the ACP conversation is important. One of the possible barriers may be related to time constraints in these practices and therefore, implementing interdisciplinary models where realization of ACP initiatives may be more tenable. | Page 23, lines 544-554 |
|  | References  Titles of cited articles are alternately written with sentence style and with headline style and the name of the journal is likewise written out in full, with and without capitalisation and written in their abbreviated form. Please adhere to the author guidelines for these two issues.  I would suggest that you consider changing reference 33 to another one. There must be a written references where this can be found. | References are now consistently formatted appropriately per journal requirements.  Reference 33 is changed as suggested. One reference was mentioned twice, its now removed. |  |
|  | Table 1  In the list of common chronic conditions you have listed MSK conditions as well as HTN without giving the abbreviated expression while you are not abbreviating coronary artery disease with CAD. In the name of logic, this would have been what I had expected. I would suggest that you write the expressions out in full since I am pretty sure that these abbreviations not are fully transparent for all potential readers.  I am also surprised to not find the very common *cerebrovascular diseases* among the chosen chronic conditions, since it is one of the most common chronic conditions globally and not least since it also is a common contributing underlying cause to dementia.  Why is there no age range for the trusted individuals? If a mean with SD have been calculated, the range must be known. | The full form of the conditions are now added to the table. Also TIs’ age range is specified.  Stroke was not identified because it was not reported as one of the diagnoses in any of the participants, however all of them had high vascular burden |  |
|  | Table 2  It would be good if min and max score for the participants experiences were described in proximity to the table itself or where the questionnaires are described. | The following description is added to the manuscript in the measures section:  Specific qualities of sound and video were rated using a 5-point scale on the ability to see and hear where 1 represented none of the time and 5 represented all of the time; whereas frequency of lags in video were captured as: 1 meaning, none of the time to 5 meaning all of the time. | Page 8, lines 161-164 |
|  | Table 3  Table 3 gives the impression that you have calculated the time since the trusted individuals were diagnosed with dementia. Is that what have been done? Maybe I read the table the wrong way? | We have changed the name of the predictor to bring more clarity. It is now called, “Time since dementia diagnosed in PLwD, days” Here we are hoping to see if there was any influence on PLwD and/or trusted individuals related to the time passed since the PLwd first received their diagnosis. |  |
|  | Table 4  I have difficulties understanding this table and there are several issues:  The table text states that categories and themes are given in the table. However the word theme is not present anywhere in the table.  I the first column it I stated that five categories were established but I struggle to find them. Is *Acceptability* one category? And is *Participants’ experiences of engaging in VYV virtually?* In that case I find two categories.  I also have difficulties with understanding the last column with Description of the subcategories. How is “VYV as a means to engage in ACP process” a description of the subcategory “Breaking the ice”? The same applies for all description in this column. | Your are right, there are two broad categories and each of them have subcategories. We have now made clarifications in the text and provided more clarity to the description of each subcategory. |  |
|  | Table S1  It is a bit difficult to understand which of the quotes belongs to which subcategory since no vertical lines that guides the reader, or extra free space is present. The quotes adds no value if the reader will have to guess which category they illustrate. | We have now added lines to give clear delineation for the ease of the readers. |  |
|  | Language  Line 34 -35 contains an “any” each, that is two in a row in that sentence. | Thank you for pointing this out, it is now corrected |  |
|  | Deposition of data  In the part of the submission that I have got access to it is stated that all data are fully available without restriction but I cannot find any information about where the data are deposited. | The data will be available upon reasonable request, this was made more clear at the time of resubmission. |  |
|  | Guidelines for studies  No guidelines for publication is referred to in the manuscript and no guideline document is included in the part of the submission that I have access to. | To ensure quality, rigor, transparency, and completeness, the CONSERVE (CONSORT and SPIRIT Extension for RCTs Revised in Extenuating Circumstances) was used. The CONSERVE checklist allowed us to report modifications to the study due to extenuating circumstances related to the COVID-19 pandemic. Additionally, the template for intervention description and replication (TIDieR) checklist was used to describe details of the VYV intervention for future replication. These are mentioned in the other manuscript describing the feasibility of the study. This is now added in this paper too in the rigour section. | Page 9, lines 189-192 |
